# Associations between street connectivity and active transportation

**DOI:** 10.1186/1476-072X-9-20

**Published:** 2010-04-23

**Authors:** David Berrigan, Linda W Pickle, Jennifer Dill

**Affiliations:** 1 ARP, DCCPS, National Cancer Institute, Bethesda, MD, USA; 2 SRP, DCCPS, National Cancer Institute, Bethesda, MD, USA; 3 StatNet Consulting, Gaithersburg MD and The Pennsylvania State University, University Park PA, USA; 4 College of Urban and Public Affairs, Portland State University, Portland OR, USA

## Abstract

**Background:**

Past studies of associations between measures of the built environment, particularly street connectivity, and active transportation (AT) or leisure walking/bicycling have largely failed to account for spatial autocorrelation of connectivity variables and have seldom examined both the propensity for AT and its duration in a coherent fashion. Such efforts could improve our understanding of the spatial and behavioral aspects of AT. We analyzed spatially identified data from Los Angeles and San Diego Counties collected as part of the 2001 California Health Interview Survey.

**Results:**

Principal components analysis indicated that ~85% of the variance in nine measures of street connectivity are accounted for by two components representing buffers with short blocks and dense nodes (PRIN1) or buffers with longer blocks that still maintain a grid like structure (PRIN2). PRIN1 and PRIN2 were positively associated with active transportation (AT) after adjustment for diverse demographic and health related variables. Propensity and duration of AT were correlated in both Los Angeles (r = 0.14) and San Diego (r = 0.49) at the zip code level. Multivariate analysis could account for the correlation between the two outcomes.

After controlling for demography, measures of the built environment and other factors, no spatial autocorrelation remained for propensity to report AT (i.e., report of AT appeared to be independent among neighborhood residents). However, very localized correlation was evident in duration of AT, particularly in San Diego, where the variance of duration, after accounting for spatial autocorrelation, was 5% smaller within small neighborhoods (~0.01 square latitude/longitude degrees = 0.6 mile diameter) compared to within larger zip code areas. Thus a finer spatial scale of analysis seems to be more appropriate for explaining variation in connectivity and AT.

**Conclusions:**

Joint analysis of the propensity and duration of AT behavior and an explicitly geographic approach can strengthen studies of the built environment and physical activity (PA), specifically AT. More rigorous analytical work on cross-sectional data, such as in the present study, continues to support the need for experimental and longitudinal study designs including the analysis of natural experiments to evaluate the utility of environmental interventions aimed at increasing PA.

## Background

Physical activity contributes to health through its direct effects on disease risk as well as its indirect effects via contributions to weight loss and weight maintenance. These benefits have been comprehensively reviewed in a recent report from the US Physical Activity Guidelines Advisory Committee [1]. However, there is evidence to indicate that there is an epidemic of sedentary behavior in the developed world [2]. Recent results based on objective measurement of physical activity using accelerometers in the US and Sweden suggest that the prevalence of adherence to PA guidelines is even lower than that indicated by studies based on health surveys, with only about 5% of US and Swedish adults adhering to physical activity guideline recommendations of 30+ minutes of moderate or greater intensity PA five or more days per week [3,4].

Walking and bicycling for transportation and/or leisure are a major form of physical activity worldwide [5], and such activities can meet recommendations for physical activity [6]. Individual interventions to increase walking/bicycling are expensive and have seldom been implemented at the population level. Furthermore, campaigns aimed at changing behavior absent environmental change may have small or poorly maintained effects [7-9]. Thus, there is considerable interest in the potential for understanding and improving the active transportation (AT) environment as a way to increase walking and bicycling for health and to alter mode share away from automobiles towards AT, a goal thought to have environmental, energy, and potentially social benefits [10,11].

Street connectivity is one major environmental or 'built environment' feature that could have direct or indirect influences on AT. Street networks that are more connected are thought to increase walkability and those that include longer blocks, fewer intersections, and more dead-ends are argued to be less conducive to walking. Direct effects of connectivity could include ease of walking from place to place and the aesthetic correlates of more connected networks. Indirect effects of connectivity are often associated with the association between destinations and connectivity. Connectivity creates more and shorter routes to such destinations [12-15].

Diverse studies have examined the association between various measures of street connectivity including block length [16], block size [17-19], intersection density [18,20], percent four way intersections [16,21]; street density [22,23]; connected intersection ratio [19,24], and link node ratio [25]. Grid block and path length characteristics and derived indices such as the alpha and gamma index (see below) have also been reported and analyzed in relation to pedestrian behavior and mode choice [14,26-29]. Many, but not all of these studies find positive associations between measures of connectivity and AT or leisure walking. Recent papers have also called attention to the fact that many of these positive associations are weak, even when statistically significant [30-33]. It also seems likely that such measures are correlated with one another and therefore it is not obvious what specific recommendation about street network design arise from this body of work. The **first goal **of this paper is to extract multiple measures of street connectivity in a single study and try to identify the underlying factors describing street networks that are associated with active transportation via walking and bicycling.

A **second goal **of the paper is to add a geographic perspective to the analysis of associations between street connectivity and AT. Past studies of street connectivity have largely or completely ignored the fact that respondent environments are distributed spatially and likely to be correlated with one another over some (unknown) spatial scale. Sometimes this issue has been addressed by comparing specific neighborhoods selected to differ with respect to urban form and other variables and separated geographically [22]. In this paper we explicitly explore the effects of geography by including spatial random effects in our analysis of associations between street connectivity and active transportation behavior.

The **third goal **of the paper is to examine propensity and duration of AT separately. Behavioral traits such as leisure time walking and bicycling, AT or other forms of physical activity have at least two components, the probability or propensity to engage in the behavior and the duration of the behavior in the people who are active (we acknowledge that other components such as intensity and affect are not included here). Many past studies of built environment and walking have analyzed propensity and duration separately; thus we aim to illustrate the use of a multivariate distribution with a binary component for walking propensity and a log normal component for walking duration. This approach should provide more statistical power to detect covariates associated with both aspects of AT.

To address these goals we analyzed street connectivity and its association with AT using a large spatially identified data set collected as part of the 2001 California Health Interview Survey. Street connectivity represents a major class of environmental variables of great interest to health geographers because they are potentially correlated with multiple health behaviors and organized over diverse spatial scales.

## Methods

Additional detail concerning the survey and variables analyzed here are presented in Huang et al. 2009 [34]. This study is based on a subset of data from the 2001 California Health Interview Survey (CHIS). This large (N = 55,428 households) random digit dial telephone survey in California is administered in seven languages (English, Spanish, Mandarin, Cantonese, Vietnamese, Korean and Khmer) and had a response rate, based on the American Association for Public Opinion Research equation RR4 [35], of 43.3% with a cooperation rate of 63.7% (weighted to account for the sample design) and 77.1% (unweighted).

We studied residents of San Diego and Los Angeles counties where over 70% of survey respondents supplied the name of the nearest intersection to their residence (In LA County, 8728/12196 = 71.5%, and in SD County 1952/2672 = 73%). These addresses were geocoded to represent the location of each respondent for purposes of this analysis. After exclusion of respondents with missing or invalid data, 8506 respondents from LA and 1883 respondents from SD were used in the analysis. These two counties were the only ones with nearest intersection data available in CHIS 2001.

The paper has two sections. In the first, we characterize street connectivity based on GIS-derived measures from buffers around the nearest intersections to respondents homes. In the second section we used a combination of CHIS variables, Census data, and the street connectivity data in a model-based analysis to explore the relative contributions of street connectivity and other variables to active transportation (AT).

### Contextual and connectivity variables

We compiled street connectivity and two density-related variables using circular buffers (areas around a point) of radius 0.5 km surrounding each respondent's location (nearest intersection to home). These buffers were defined using TIGER map files from the 2000 U.S. Census Bureau and implemented with GIS software (ArcView, ESRI, Inc.). Data concerning population and employment density and characteristics of the street network for each buffer were then calculated at the census tract or census block (administrative units that are nested within census tracts) level.

Population density within a buffer was generated by downloading US Census data at the census block level. Each half-kilometer buffer usually overlapped more than one census block. We assumed that population density is uniform within each census block and assigned a portion of the population within the census block to the buffer based on the area of the census block within the buffer. For example, if a buffer covers half of a census block, half of the census block's population is assigned to that buffer, in addition to the population in census blocks that were completely within the buffer. The total population in the buffer was then divided by the area (0.785 square kilometers). Employment density data were generated using data from the metropolitan planning organization for each area - the Southern California Association of Governments (SCAG) for Los Angeles and the San Diego Association of Governments (SANDAG) for San Diego. Each agency provided total employment data by census tract for the year 2000. The method to calculate employment density was identical to that of population density, except that because of census data availability, we used tracts instead of blocks. Therefore, the variance associated with population and employment densities are likely to differ in this study.

For our measures of street connectivity, we first extracted or calculated values for nine variables for each buffer. Later we used principal components analysis (see results) to reduce the number of variables used in our analysis of variance. Variables included: 1) Link/Node Ratio, the link/node ratio is the total number of links divided by the total number of nodes. All nodes are included, meaning intersections and the ends of cul de sacs and dead-end streets. A higher ratio = higher connectivity. Links are defined as street segments and nodes as intersections or dead ends. 2) Intersection Density, intersection density is the number of real nodes (nodes that are at 4-way or 3-way intersections, not the end of cul de sacs) divided by the buffer area (0.785 sq. km.). A higher density = higher connectivity. 3) Street Network Density, the street network density is calculated by summing the lengths of all the links within the buffer (the total network distance within the buffer, ignoring the number of lanes on a road) and dividing by the area of the buffer (0.785 sq. km.) (Note buffer size choice was based on our expert opinion, budget constraints precluded analysis of more buffer sizes). The portion of a street (link) that continued outside the buffer was not included. A higher density = higher connectivity. 4) Connected Node Ratio, connected node ratio (CNR) is the number of real nodes divided by the total number of all nodes. If all the nodes in a buffer were at 4-way or 3-way intersections, the CNR would be 1.0. A higher ratio = higher connectivity (maximum = 1.0). 5) Block Density, block density is the total number of Census blocks within a buffer divided by the area of the buffer (0.785 sq. km.). Census block boundaries generally coincide with streets and are consistent with a block defined by the area within connecting streets. If a portion of a block was outside a buffer, only the area of the block within the buffer was included. A higher density = higher connectivity. 6) Average Block Length, the average block length is the average length of the links that are completely or partially within the buffer. For links (blocks) that continue outside the buffer, the entire length of the link is included in the calculation. Truncating the link at the buffer boundary would have reduced the length of the block artificially. A higher average length = less connectivity. 7) Median Block Length, median block length was calculated in the same manner as average block length. A higher median length = less connectivity

The eighth variable was the Gamma index, the ratio of the number of links in the network to the maximum possible number of links between nodes. The maximum possible number of links is expressed as 3 * (# nodes - 2) because the network is abstracted as a planar graph. In a planar graph, no links intersect, except by nodes [28]. Values for the gamma index range from 0 to 1 and are often expressed as a percentage of connectivity, e.g. a gamma index of 0.54 means that the network is 54 percent connected. Only links that are completely within the buffer were included. This was because every link must have a node on each end. If links were truncated at the buffer, there would be no node. In addition, only the nodes that intersect with these links were included. Gamma was only calculated for buffers with three or more nodes. All the locations with the number of nodes less than 3 were treated as missing (3 points in SD and 6 points in LA). A higher value = higher connectivity (maximum = 1.0).

The ninth variable was the Alpha index. The alpha index uses the concept of a circuit - a finite, closed path starting and ending at a single node. The alpha index is the ratio of the number of actual circuits to the maximum number of circuits and is equal to:

Values for the alpha index range from 0 to 1. As with gamma, only links that are completely within the buffer were included and only the nodes that intersect with these links were included. Alpha can not be calculated if the number of nodes in a buffer is less than three or the number of nodes is equal to or greater than the number of links. These cases were coded as missing data (98 points in SD and 128 points in LA). The second condition was violated more often than the first, because only links within a buffer be included. This was usually in more rural areas. A higher value = higher connectivity (maximum = 1.0)

Several of the above measures were highly correlated; 7 of the 36 possible pairs of the 9 variables had correlation coefficients above 80% (See below). Including highly correlated covariates in a regression model leads to instability of the model, so we used principal components (orthogonal rotation) and factor analysis to identify the main components of variance in this data set. This process constructed indices that explained most of the variance of the built environment across the locations and that could be used as independent predictors in the models. Similar principal components were derived from analyses considering LA and SD separately. These analyses were carried out in SAS JMP Version 8.0 (Cary, NC).

### Active transportation, demographic, and anthropometric variables from CHIS

CHIS 2001 survey data included in this study were a measure of active transportation, and multiple relevant demographic and anthropometric variables. AT was measured by asking three short questions: 1) "Over the past 30 days, have you walked or bicycled to or from work, school, or to do errands?", 2) "How many times per day, per week or per month did you do this?" and 3) "And on average, about how many minutes did you walk or ride your bike each time?". AT was analyzed either as a measure of prevalence such as yes/no (any AT or none) from the answers to the first question, or as a measure of duration such as minutes per week among walkers/bicyclists derived from the answers to the second and third questions.

Demographic and socioeconomic status (SES) variables including age, gender, race, education, and income were also extracted from the CHIS survey resource for each respondent, as were self-reported health status, immigration status and employment status. For self related health status we chose an activity related variable based on responses to the query "How much does your health limit you when climbing several flights of stairs?". Responses were on a three part scale, "Limited a lot", "Limited a little", "Not limited at all". CHIS includes a variety of other variables related to diet, tobacco and alcohol use, cancer screening practices, health care coverage; we focused on variables commonly used in past studies of active transportation. For some analyses we also used self-reported data on height and weight to obtain body mass index [BMI = Height/Weight (kg)^2^], a measure of obesity.

Geographic identifiers included latitude and longitude rounded to 0.01 degrees and Zipcode of address. Data concerning bus stops and light rail were obtained from the Los Angeles and San Diego Public transit agencies coded as present or absent within a buffer (Thanks to R. Adamski). Presence or absence of a freeway within a buffer was obtained from Tiger Line files.

### Statistical analysis

Preliminary analysis showed that the distribution of the number of minutes reported in AT was skewed and had a spike at zero, representing respondents who do not report any AT. A logarithmic transformation normalized the distribution of non-zero minutes. The importance of the potential explanatory variables was tested separately by a logistic model for the AT/no AT response and a lognormal model for the number of minutes reported by those with any AT [36]. These fixed effects models included all main effects and all possible two-way interactions at first. Non-significant (p > 0.05) interactions and then main effects were removed by a stepwise procedure.

Once the initial subset of variables and their interactions were determined, the data were analyzed by a multivariate regression, with a binary component for whether a person reported any AT and a lognormal component for the number of minutes of AT. This approach has the advantage of increased power to detect significant effects that indicate a common association with both responses. For example, if older respondents were less likely to report any AT and those who did report any AT spent less time in AT, then the combined model could estimate a single parameter for the age effect, increasing the power over that from two separate models. Another advantage of the multivariate model is that it can measure any correlation between the propensity to report AT and the length of time spent in AT in geographic areas with multiple respondents. A disadvantage of this approach is that the more complex model is difficult to apply, requiring larger sample sizes and greater computational effort to estimate its parameters than either model component separately. These difficulties are compounded by the need to account for the correlation of responses among neighbors.

Methods have been developed to analyze data that result from a mixture of two different statistical distributions. Zero-inflated Poisson (ZIP) methods, introduced by Lambert in 1992 [37], are regression models for count data with an excess number of zero responses. These models include a model component to represent the probability the dependent variable occurred in a subject. More recently, these zero-inflated mixture model methods have been extended to other types of data [38]. For example, Tooze et al. proposed a mixture model that included random effects correlation among the repeated responses of individuals [39]. This method has been applied successfully to 24-hour dietary recall data, with separate regression components for whether the respondent ate a particular food during that day and for their amount consumed of that food [40]. The probability that a person ate the food is modeled by a logistic regression model and the usual amount consumed is modeled by a normal regression model, after a suitable normalizing data transformation. This model produces a direct estimate of the correlation between the two model components but does not allow estimation of spatial correlation of the respondents, an important goal of our CHIS analysis.

We used SAS PROC GLIMMIX to implement a multivariate model that is a mixture of logistic and lognormal regression components for the probability that a person reported any AT and the amount of AT, respectively, similar to the model for dietary intake described above [[41] example 5]. Covariates found to be significant predictors of either outcome (any AT and amount of AT) were included and were initially allowed to vary by type of outcome. Those with non-significant effects, as measured by p-values of the Type III (partial) sums of squares F test greater than 0.05, were removed. If there was no significant difference in an effect between the two model components, the two parameters were replaced by a single common parameter for that effect. Covariates that were significant predictors for only one of the two counties were retained in both county models for comparability of effects. Covariates indicating gender, race and age were retained regardless of significance in order to compare effects across models and counties.

Each of the two regression components could include correlation among persons living in the same small geographic area, i.e., AT habits could be similar in small neighborhoods. Failure to account for this correlation, if it exists, violates the assumption of independent residual errors in standard regression analyses and can lead to mis-specification of the variances and covariances of model parameters, which in turn leads to mis-specification of the corresponding statistical significance. The spatial correlation in the original data can be accounted for by model covariates that explain the spatial patterns or by use of a spatial error structure for the variance/covariance matrix of a model random effect (a hierarchical analysis) or of the model residuals. For this analysis, we attempted to include covariates that would explain most of the underlying spatial pattern in AT behavior but also included a random effect to account for any remaining spatial correlation.

We did not assume that the degree of spatial correlation was identical for the two types of responses. Spatial correlation was assessed in two ways: by an exponential decay function where correlation decreased with increasing distance between respondents' addresses, and by a threshold function where responses of persons who lived within a defined neighborhood had a constant correlation but were not correlated at all with responses from outside that neighborhood. Spatial correlation for each county was assessed by using a spline approximation on a 30 × 30 cell grid, corresponding to neighborhoods approximately 2.3 miles square; smaller neighborhoods had too few respondents for stable assessment of the correlation. The threshold model was repeated with neighborhood defined by the respondents' postal zip codes.

No single statistic is available to assess how well mixed effects models fit because of the complexity of the likelihood in the presence of random effects. We compared values of the generalized chi-square statistic for goodness-of-fit and checked the final models by rerunning their fixed effects equivalents separately to calculate the Hosmer-Lemeshow statistic [42] for the logistic component and the likelihood ratio statistic for the lognormal component. Residuals were examined and variograms were plotted and compared for the original and residual data. Distances for the variogram calculations were Great Circle distances based on the geocoded locations.

The spatial and non-spatial models cannot be compared directly because of the default likelihood approximation used by SAS PROC GLIMMIX for random (spatial) effects models. Therefore we attempted to rerun the final models on a more powerful LINUX PC to obtain exact likelihood results. The local neighborhood spatial models did not converge, required more computer memory than was available or produced an invalid variance/covariance matrix. The zip code threshold models did converge using adaptive quadrature integral approximation methods. Because of the computational difficulties in optimizing the exact likelihoods, particularly for the larger LA sample, the results in this paper are the pseudo-likelihood (Restricted Maximum Likelihood) results, unless otherwise specified.

The computational difficulties involved in estimating parameters in models where the variances/covariances are unknown, as is the case for spatial models, are well documented [[43], Chapter 9]. Inclusion of random effects or the need to estimate the covariance parameters requires use of an iterative estimation procedure, i.e., there is no exact solution to the optimization equations. Assessment of convergence, as reported above, is essential for any of these models, as it gives some assurance that the results are reliable. We addressed this problem by using a well-tested commercial software program [34] for the iterative parameter estimation process and by screening covariates and their interactions carefully to develop a parsimonious model to improve model stability. Finally, we compared results for several types of models (fixed and random effects, separate and joint propensity and duration models), with several subsets of covariates and at different geographic scales, looking for consistent effects.

The joint model of propensity and duration is complex but allows information about one type of outcome (propensity or duration) to aid in predicting the other, in theory providing a more robust approach than analyses treating propensity and duration separately or simply using logistic regression with zero or zero + low levels of activity as one of the categories in the dependent variable.

## Results

This paper concerns the association between active transportation as measured by self-reported levels of active transportation (AT) and independent variables including street connectivity, demographic characteristics of respondents, and a set of contextual variables related to neighborhood SES and transit access.

Respondents from the study counties, LA (n ~8,500) and SD (n ~1,900), have moderately similar characteristics compared to the entire state of California [34]. There are some differences between California and the US as a whole, between California and LA/SD, and between the two counties. The LA/SD sample is more racially/ethnically diverse than California as a whole (Table 1). Compared the United States, LA and SD combined and the entire state of California are more racially/ethnically diverse, younger, have lower income, and have more immigrants and more college graduates and residents who did not graduate from high school **(see also **[34]. The two counties are similar in age structure, but San Diego has a much higher percentage of non-Hispanic Whites, a lower percentage of people earning less than 100% of the poverty level, and a lower percentage of people with less than a high school education. The percent of respondents reporting any active transportation in LA was higher than in SD (42.0% vs. 36.1%), whereas the average duration of active transportation LA and SD were similar (84 vs. 80 minutes per week).

**Table 1:** Demographics of subject counties (based on respondents only), California (from CHIS 2001) and the entire USA (from the 2001 National Health Interview Survey [34]).

Variable	Los Angeles*	San Diego*	Combined*	California*	USA
Area (sq.km)	12,308	11,721	24,039	410,000	9,631,000
Population	9,662,000	2,813,000	12,475,000	34,400,000	285,000,000
Sample size	8,547	1,891	10,438	56,270	69,244
**Gender (%)**					
M	50.7 (0.4)	50.3 (0.7)	50.6 (0.3)	48.9 (0.0)	47.9 (0.1)
F	49.3 (0.4)	49.7 (0.7)	49.4 (0.7)	51.1 (0.0)	52.1 (0.1)
**Race/ethnicity (%)**					
Non-Hispanic White	39.4 (0.4)	61.2 (0.8)	44.6 (0.3)	50.2 (0.0)	73.6 (0.4)
Non-Hispanic Black	9.9 (0.2)	5.1 (0.5)	8.8 (0.2)	5.9 (0.0)	11.2 (0.3)
Hispanic	38.2 (0.4)	22.8 (0.8)	34.5 (0.4)	29.3 (0.0)	10.6 (0.2)
Other	12.5 (0.3)	10.8 (0.6)	12.1 (0.2)	14.5 (0.0)	4.6 (0.2)
**Age**					
18-39	47.6 (0.4)	47.3 (0.8)	47.5 (0.4)	45.7 (0.0)	39.6 (0.3)
40-59	33.7 (0.4)	32.8 (0.7)	33.5 (0.4)	34.9 (0.1)	38.1 (0.3)
60+	18.8 (0.3)	19.8 (0.8)	19.0 (0.3)	19.4 (0.1)	22.3 (0.2)
**Income (% of poverty level)**					
< 100	17.2 (0.5)	10.6 (1.0)	15.6 (0.4)	15.7 (0.2)	10.0 (0.2
100-200	21.7 (0.6)	21.0 (1.2)	21.5 (0.6)	20.4 (0.3)	16.8 (0.3
200-300	14.0 (0.5)	14.2 (1.0)	14.0 (0.5)	14.2 (0.2)	17.2 (0.3
300+	47.2 (0.7)	54.2 (1.5)	48.8 (0.7)	49.7 (0.2)	56.0 (0.4
**Education (%)**					
<HS	20.9 (0.7)	14.6 (1.1)	20.9 (0.5)	21.4 (0.1)	16.7 (0.2)
HS graduate	23.2 (0.5)	25.0 (1.2)	23.2 (0.5)	23.3 (0.1)	30.5 (0.3)
> HS	55.9 (0.7)	60.4 (1.3)	55.9 (0.6)	55.3 (0.2)	52.8 (0.4)

### Street connectivity

We extracted information concerning nine measures of street connectivity. Values of these measures are typical for large urban areas in the western and southern US (Table 2). The nine measures of street connectivity show a complex pattern of correlation (Table 3). Measures of block length are positively correlated with each other but negatively correlated with intersection and street density. Not surprisingly, there were strong positive correlations between alpha and gamma and measures of node characteristics, link node ratio and connected node ratio. This correlation structure made data reduction seem desirable, but inspection does not make it obvious if one or two of the existing variables could adequately represent the variation present in these measures of street connectivity (Table 2, 3). Therefore we chose to perform principal components analysis to try to identify underlying axes or factors accounting for variation in the data. Two factors account for 84% of the observed variance, with the third and fourth axes accounting for only 7 and 3% of the total variance (Table 4). Principle component one (PRIN1), accounting for 55% of the variance, showed positive loadings on all the variables except for negative loadings on the two measures of block length. Thus, it represents neighborhoods with relatively short blocks and relatively higher intersection density and proportion of 4 way intersections. The second axis (PRIN2), accounting for an additional 29% of the variation, had positive loadings on street length and negative loadings on intersection density, street density, and block density. Thus it represents buffers with longer block lengths. Measures of node characteristics are still loading positively, thus these are connected neighborhoods, but with longer blocks reducing the density of intersections and blocks. Analysis of these two variables preserves most (84%) of the variation present in our data, but removes several computational difficulties by replacing 9 highly correlated predictor variables with two independent ones.

**Table 2:** Means and standard deviations for connectivity variables in Los Angeles (n = 8542) and San Diego (N = 1942) counties.

	Los Angeles		San Diego	
Variable	Mean	**S.D**.	Mean	**S.D**.
Link Node Ratio	1.828	0.263	1.627	0.268
Connected Node Ratio	0.863	0.123	0.751	0.133
Intersection Density	47.05	21.72	39.25	18.28
Street Density	11.96	2.853	10.16	3.179
Block Density	37.51	20.49	29.01	19.36
Average Block Length	0.168	0.053	0.181	0.110
Median Block Length	0.160	0.055	0.156	0.089
Alpha*	0.163	0.084	0.113	0.090
Gamma*	0.449	0.056	0.416	0.060

N = 8414 and 1852 for alpha and 8536 and 1911 for gamma in Los Angeles and San Diego counties respectively

Note values for all variables are significantly different between counties (p < 0.0001).

**Table 3:** Spearman correlations amongst street connectivity variables

	LNR	InD	CON	STD	Gam	Alpha	BD	MedB	AVbL
Link Node Ratio	1.000	0.151	0.884	0.406	0.817	0.772	0.306	0.382	0.108
Intersection Density		1.000	0.360	0.865	0.424	0.492	0.854	-0.491	-0.591
Connected Node Ratio			1.000	0.550	0.832	0.830	0.414	0.155	-0.102
Street Density				1.000	0.573	0.652	0.786	-0.339	-0.556
Gamma					1.000	0.977	0.539	0.086	-0.125
Alpha						1.000	0.588	-0.047	-0.289
Block Density							1.000	-0.362	-0.484
Median Block Length								1.000	0.766
Average Block Length									1.000

**Table 4:** Principle Components analysis of street connectivity variables

Eigenvalue	4.958	2.596	0.628	0.298
Percent	55.092	28.849	6.982	3.311
Cum Percent	55.092	83.941	90.922	94.234
				
Link Node Ratio	0.3054	0.4172	-0.1094	-0.2381
Intersection Density	0.3453	-0.3079	0.4147	-0.0712
Connected Node Ratio	0.3603	0.2914	-0.1914	-0.3613
Street Density	0.3911	-0.1710	0.2638	-0.4341
Gamma	0.3870	0.2503	-0.1512	0.4498
Alpha	0.4087	0.1694	-0.2494	0.3422
Block Density	0.3624	-0.2037	0.4533	0.3491
Median Block Length	-0.1040	0.5307	0.4348	-0.2629
Average Block Length	-0.2140	0.4501	0.4797	0.3300

### Spatial characteristics of the data

**Figure 1:**
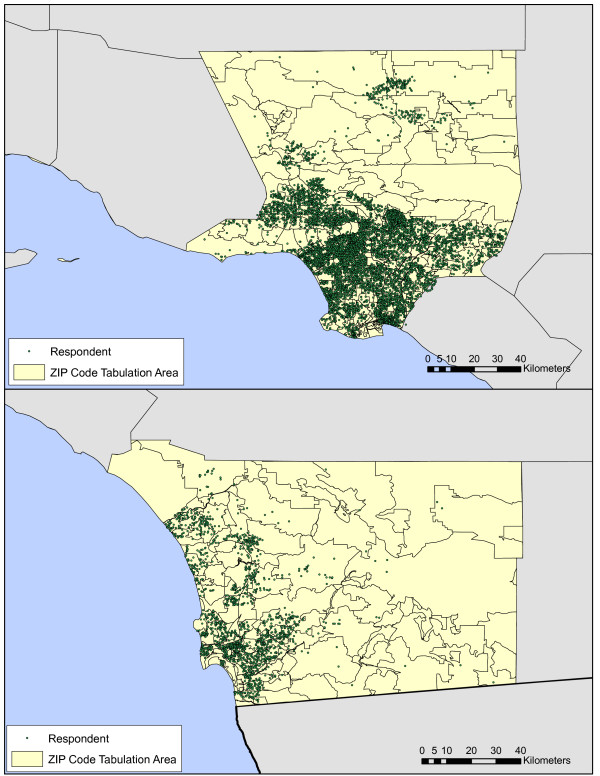
**a, b. Approximate locations of respondents in Los Angeles (a) and San Diego (b) counties**.

**Figure 2:**
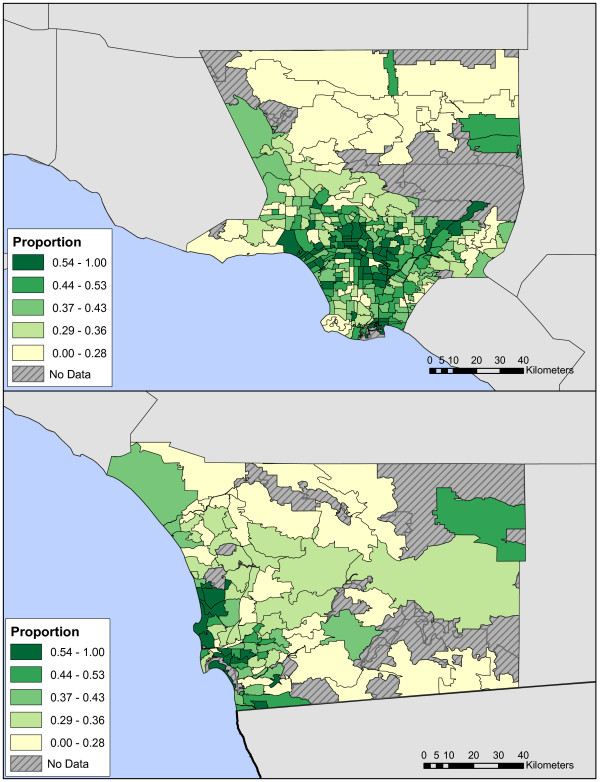
**a, b. Choropleth maps of % reporting any active transportation by zip code in Los Angeles (a) and San Diego (b) counties**.

**Figure 3:**
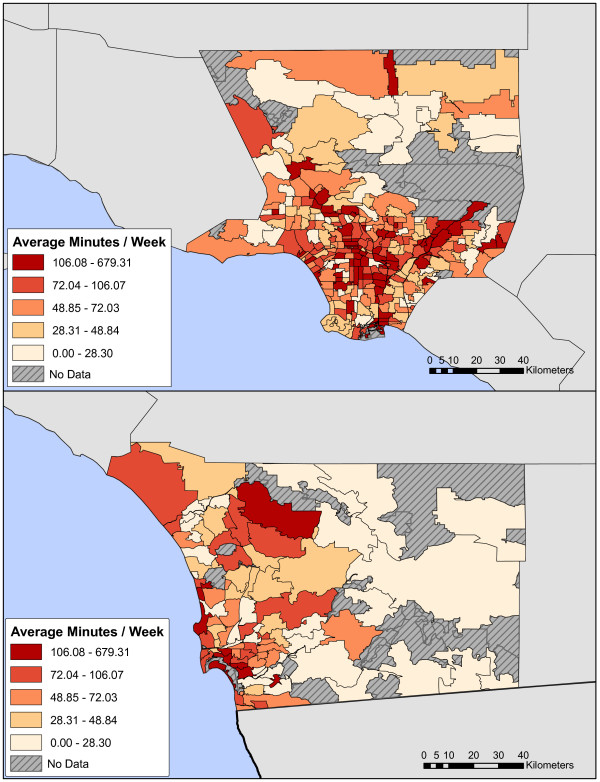
**a, b. Choropleth maps of mean active transportation duration (minutes per week) by Zipcode Tabulation Area's (ZCTA's) in Los Angeles (a) and San Diego (b) counties**.

Several figures illustrate spatial characteristics of respondents in LA and San Diego. Respondent density is roughly proportional to population density (Fig 1a, b) with concentrations, for example, of respondents in the cities of San Diego, downtown Los Angeles, Santa Monica, and Long Beach. Choropleth plots of percent reporting any AT by zip code (Fig 2a, b) and average duration of AT in respondents with any AT (Fig 3a, b) illustrate regional heterogeneity in the prevalence of AT. Huang et al. [34] used spatial scan statistics to identify clusters of elevated or reduced AT prevalence. In this paper we take the complementary approach of examining the impact of pre-selected candidate determinants of AT prevalence and duration simultaneously in an analysis that accounts for spatial clustering using random effects.

**Figure 4:**
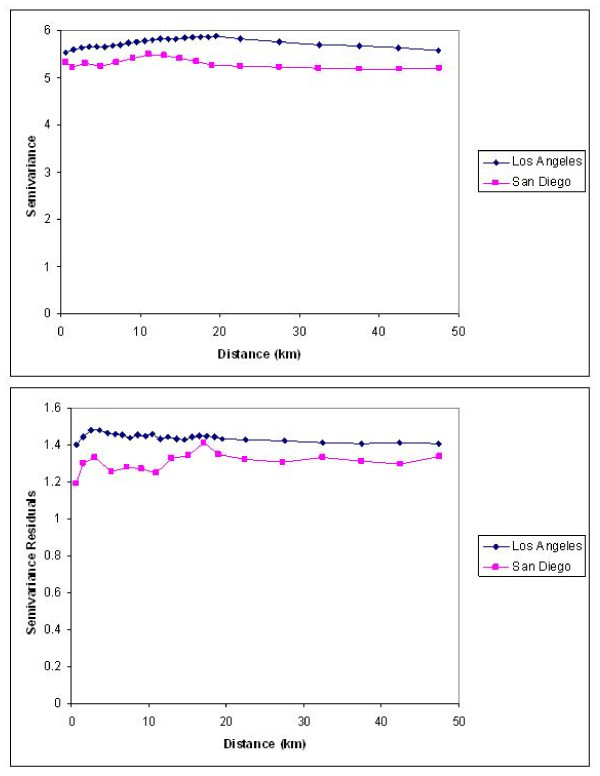
**a, b. Semiovariograms illustrating the level of spatial autocorrelation for AT duration (logarithm of number of minutes) in Los Angeles (a) and San Diego (b) counties**.

Semivariograms were used to determine the scale of spatial autocorrelation [43]. These graphical analyses indicated that the correlations within each county were stronger than the correlations of responses between counties, so Los Angeles and San Diego were analyzed separately. The semivariograms also suggested that the spatial correlation was limited to respondents who lived within 10 (SD) to 20 (LA) kilometers of each other (Fig 4a). Therefore spatial correlation for each county was assessed by using a spline approximation on a 30 × 30 cell grid, corresponding to neighborhoods approximately 2.3 miles square; smaller neighborhoods had too few respondents for stable assessment of the correlation. The threshold model was repeated with neighborhood defined by the respondents' postal zip codes. These larger geographic units masked very localized spatial correlation as evident in the semivariograms, but had the advantage of large numbers of respondents in most areas with which to assess the correlation between the propensity to report AT and the amount of AT.

To explore the spatial scale of street connectivity and AT, multivariate analyses were run at 2 geographic levels: zip code (large) and latitude/longitude (small, rounded to 0.01 degrees); there were 277 unique zip codes and 2463 unique latitude/longitude combinations in LA, 91 zip codes and 856 latitude/longitude combinations in SD. On average, there were 31 people/zip code and 2.5 people/latitude-longitude in LA and 21 people/zip code and 1.5 people/lat/long in SD. The square latitude/longitude "neighborhood", rounded to 0.01 degrees, has a diameter of about 0.6 miles, close to the buffer size (circle radius = 0.31 miles).

### Model results

The final sets of covariates (Additional File 1) fit the observed data well according to the logistic goodness-of-fit fixed effects test (Hosmer-Lemeshow chi-square statistic = 9.04, p = 0.33 in LA and 12.28, p = 0.14 in SD) and a residual analysis of the lognormal fixed effects model of duration. Inclusion of neighborhood characteristics (see Additional File 1) was a significant improvement over the fixed effects model with only individual characteristics in LA (likelihood ratio chi-square statistic = 101.94, df = 19, p < 0.0001) but not in SD (likelihood ratio chi-square statistic = 12.64, df = 19, p = 0.856). The fixed effects logistic model of propensity to report AT showed no over-dispersion, suggesting that the decision to use AT was made independently by people within a neighborhood. In contrast, the observed semivariogram of the logarithms of duration of AT suggested a small spatial correlation within 10 (SD) to 20 (LA) kilometers, necessitating a spatial model (Fig 4a) and a spatial resolution below the observed level of spatial correlation.

The spatial neighborhood models, i.e., random effects models with local neighborhood effects, were fit to propensity and duration of AT separately and by a combined multivariate model. Although the spatial random effect estimates were not significantly greater than 0, the multivariate (joint) local neighborhood model seems justified by a smaller sum of squared errors, particularly in SD (generalized chi-square/df in LA = 1.00 for logistic, 1.44 for lognormal, 1.45 for multivariate with a common spatial effect, 1.43 for multivariate with local neighborhood spatial effect; generalized chi-square/df in SD = 1.03 for logistic, 1.39 for lognormal, 1.43 for multivariate with a common spatial effect, 1.39 for multivariate with local neighborhood spatial effect).

An additional justification for the multivariate model was that there were common covariate effects for most of the main effects, i.e., most of the main effects impacted propensity and duration of AT to approximately the same degree (Additional File 1). This was particularly true in SD, probably due to the smaller sample size there and the resulting lower power to detect differences in effects between the two model components. The use of common effects gives greater power than either of the separate models to detect a significant effect. Also, the multivariate model can account for the correlation between the percent who reported AT and the mean number of minutes walked; e.g., the observed Pearson correlations in zip codes with more than 1 respondent were 14.20% (p = 0.02) in LA and 49.1% (p < 0.0001) in SD. This reinforces the importance of our effort to model the propensity and amount of AT jointly.

Additional File 1 gives the joint model results for Los Angeles and San Diego counties respectively. This table, reflecting the model's complexity, requires some explanation. The magnitude of some associations were the same for both propensity and duration of AT; these regression coefficients and corresponding p values that test the statistical significance of the covariate (not just a single category of the covariate) for predicting AT are shown in the columns labeled "Common coefficients for duration and propensity". Some covariates had a different association with duration compared to propensity, so these regression coefficients were estimated separately by the model and are shown in the columns for duration and propensity, respectively. Thus, results for a covariate and its categories, if any, will be shown in either the "common coefficients" column or in the duration and propensity columns, but not both. Exceptions to this format are for the age effect by poverty level and for working status by race due to the presence of interactions of these effects in the model. We have chosen to display the stratified coefficients, e.g., a coefficient for the age effect for each category of poverty, rather than showing the main effect and interaction regression coefficients separately, requiring the reader to calculate the combined effects. As a result, there are two sets of p values for these stratified effects: the usual F test p value is shown in the duration and propensity columns, but an extra p value is shown that represents the significance of the difference between the stratified effects and the referent category effect. For example, the age effects for poverty levels no greater than 200% of the federal poverty level were highly significant compared to the referent level (300+%) but there was no difference between the age effect for people with incomes 201%-300% and over 300% of the federal poverty level.

In general, we emphasize p-values rather than the values of regression coefficients. This seems appropriate because the variables considered in this study are measured on many different scales. Combined consideration of regression coefficients and statistical significance of the variables examined in Additional File 1 should allow the readers to make their own judgments concerning the relative importance of the many variables examined in our analysis. Consideration of the mean values for the connectivity variables and levels of AT can also provide information about the magnitude of the associations observed here.

A variogram of the model residuals (Fig 4b) still showed some spatial autocorrelation, i.e., there was still a small association between neighborhood (within 3 km) and the duration of AT that was unexplained by the sociodemographic and built environment neighborhood measurements. Separate covariances for the logistic and lognormal components of the multivariate model could be estimated for the latitude/longitude model, but not for the zip code model. The zip code model with separate effects for the 2 model components would not converge. That is, a more complex covariance structure, i.e., one with separate spatial effects for each of the two model components, could be detected at the smaller area level compared to the larger zip code level model. This suggests that zip code areas are too large to capture the spatial variation in AT.

### Common model effects across SD/LA and latitude/longitude and zip code

There were a number of common effects across the two counties and smaller spatial units, latitude/longitude and zip code (Additional File 1, Zip code effects not shown). 1) Gender had no association with AT at any spatial scale. 2) Age had nearly the same association with amount of AT for all 4 models- older respondents had slightly more minutes or AT (approximately 1% more per year of age); however, older age had the reverse association with propensity to report AT for all 4 models - older ages were less likely to report AT (approximately 1% less per year of age). In LA, older residents with an income less than 200% of the federal limit were less likely to report AT and tended to have less AT than residents with a higher income. 3) There is a trend for less reported AT among those with more health limitations; an even stronger association was seen between propensity to report AT than amount of AT in LA; no significant difference could be detected in SD. 4) Hispanics are more likely to report AT than Whites, but this is not significant in SD. 5) People who were working were much less likely to report AT and tended to report less AT; this association was attenuated in Blacks in LA.

### Difference between SD and LA

San Diego and Los Angeles differed in a number of ways. 1) There is no significant effect of BMI, except for the obese in LA and overall in LA for the zip code model. 2) Birth outside the US had a significant positive effect on propensity to report AT and amount of AT, but is stronger in SD. 3) Education had a significant effect in LA, not SD, and the LA effect varies for binary and lognormal components (Additional File 1). 4) There was a strong, but nonlinear across categories, effect of population density on both propensity and amount of AT in LA, not SD. 5) There was a stronger effect of poverty level in LA than in SD for both outcomes (lower income associated with more AT). Only the 100-200% of poverty level has a significant effect in SD and no trend is evident across categories. 6) There was no difference between Blacks and Whites in SD but in LA Blacks who work are more likely to report any AT and more AT. Among higher educated residents of LA, Blacks were less likely to report AT and had less AT than other racial/ethnic groups. 7) People in SD who had lived in the US longer tended to report less AT (propensity and distance).

### Differences between local neighborhood and Zip Code models

Comparison of the AIC statistics for the models that converged using maximum likelihood estimation methods suggested that there was no advantage to the zip code threshold model over a simple fixed effects model, i.e., one that ignores any spatial autocorrelation in the data (AIC in LA = 22352 for zip code model, 22348 for fixed effects model; AIC in SD = 4501 for zip code model, 4497 for fixed effects model; lower values are better). The latitude/longitude models would only converge using a linearizing approximation to the maximum likelihood, so that no AIC statistics are available for comparison. However, these models did converge and provided spatial autocorrelation estimates for both components of the model (propensity and duration), suggesting that any spatial correlation of AT was at a very local geographic scale. There were a few differences in covariate effects between the Latitude/Longitude and Zip Code models (Not Shown). Employment density was not at all significant for predicting amount of AT in SD at the latitude/longitude level, but is a significant predictor of propensity to report AT at the zip code level (lower density was associated with less AT); results for LA were similar for both geographies. In places with more connected streets (PRIN1), a higher percentage of respondents reported AT in both LA and SD in LA there was an even stronger effect for propensity to report AT than for amount of AT, but both were significant.

### Built environment influences on active transportation

Residents of places with more connected streets and short blocks (PRIN1) were more likely to report AT in Los Angeles (p = 0.015) but the positive association of PRIN1 with duration of AT was not significant (p = 0.08). In San Diego, the association was significant for both propensity and duration (p = 0.0019). The second measure of street connectivity (PRIN2) had a small but non-significant association with AT in both Los Angeles (p = 0.0591) and San Diego (p = .1227). PRIN1 appeared to be normally distributed and had means and standard deviations of 0.26 (2.1) and -1.2 (2.3) for LA and SD respectively; PRIN2 had mean 0.095 (1.6) and -0.44 (1.7) for LA and SD. Log transformed AT minutes for respondents with any AT were 4.54 (S.D. = 1.2) for LA and 4.55 (S.D. = 1.2) for SD, or 93.7 and 94.6 minutes respectively.

Residents in SD latitude/longitude level neighborhoods with a bus stop were significantly more likely to report AT, but their duration was less. There was a common positive association of bus stops with AT in LA local neighborhoods for both outcomes, but this was not significant. There was no association of bus stops with AT in zip code areas. Despite the pedestrian unfriendliness of freeways, Los Angeles areas with freeways had residents who were more likely to report AT and had more AT. Conversely, the presence of bus routes was negatively associated with both outcomes in Los Angeles. Note that the SD zip code model does not include bus stops, freeways, bus routes or rail. Because of the smaller sample size in SD than LA, fewer covariates could be included in the SD model in order to obtain model convergence. These particular covariates were excluded because they were not at all significant in the initial propensity model for SD.

## Discussion

This study has two main results. First, diverse measures of street connectivity can be summarized by two dominant axes, one representing areas with shorter more connected blocks and the second representing areas with longer blocks, but still exhibiting a more grid like pattern. It remains to be seen whether this observation extends beyond two large cities in Southern California. Second, mixture models accounting for spatial autocorrelation indicate significant associations between measures of street connectivity and both the propensity to report AT and the amount of AT. As in past studies of built environment characteristics including street connectivity and physical activity, particularly walking [44,45], the associations between built environment remain modest. However, even small improvements in individual behaviors can have significant population health benefits. Additionally, the methodological and analytical advances implemented here are important in that they can enhance confidence in estimates of effect sizes as well as separate influences on the propensity versus duration of health behaviors generally and walking or other forms of physical activity specifically. This analytical approach could apply to diet variables, tobacco use, alcohol consumption, substance abuse, and any other behavior divisible into occurrence and dose in time or quantity.

### Street connectivity and active transportation

This study identified small but significant or near statistically significant associations between two aggregate measures of street connectivity, particularly an index representative of areas with a pattern of short blocks and a grid like structure, and active transportation (AT). This measure of connectivity (PRIN1) was more strongly associated with propensity to report AT, but was still positively associated with AT duration. These results are consistent with our recent finding that PRIN1 is elevated in clusters of active transportation identified with spatial scan analysis [34]. Without attempting to reconcile different scales for the independent variables, the magnitude of coefficients estimated for the street connectivity variables are challenging to compare directly. Consideration of mean levels of AT and means and standard deviations for PRIN1 and PRIN2 in the two counties and the coefficients reported in the supplementary file should give the reader sufficient information to think about the relative magnitude of the reported associations.

A number of past studies have also examined street connectivity and its association with different measures of AT or leisure time physical activity [10,30,46]. These studies are notable for the lack of standardization in their outcome variables, measures of connectivity and analysis approach. Handy's [30] review tabulates about 50 studies concerning built environment, AT and physical activity. More such studies have appeared since her review, including a review of built environment and walking [45]. Both reviews report consistent associations between transportation walking and density, destination distance, and land use mix, but a mix of results concerning connectivity, parks and parkland, and safety. Saelens and Handy (2008) report positive associations between route/network connectivity and walking in three of seven studies of transportation walking, zero of four studies of leisure walking, and three of six studies of general walking [45]. The remainder of the studies had null or unexpected associations. A few studies report interactions between measures of walkability and other variables such as safety or demographic characteristics - more work is needed systematically examining such interactions. Another recent study reports positive associations between density and travel walking and positive associations between large block sizes and leisure walking [31,32]. Adoption of standard metrics for connectivity would facilitate more specific comparisons of results and effect sizes in such studies.

### Demographic correlates of active transportation

Demographic correlates of active transportation were somewhat different than those reported in a recent national study of transportation walking based on data from the 2005 National Health Interview Survey [5]. In the US as a whole, transportation walking is more prevalent in men than women, decreases with age, is higher in black men and Asian/Native Hawaiian/Pacific Islander women, and is highest in the highest and lowest income categories and highest education category. By contrast, in Los Angeles and San Diego counties we found positive associations between age and duration of AT but negative associations for propensity to report AT, higher propensity but lower duration of AT in those with higher or lower than high school education (i.e., not just a high school diploma), and less AT working respondents. It is difficult to know if these differences are due to regional differences, the effects of including bicycling as a mode of AT, or effects of survey characteristics. NHIS is an in person survey and CHIS is a telephone survey. Both NHIS and CHIS results are based on self report.

Accelerometer based measures of overall physical activity [4] and step counts based on accelerometry [47] give somewhat different results as well. Overall physical activity declines with age, is higher in men than women and exhibits age by race/ethnic interactions. Step counts estimated by accelerometer are higher in US males than females; US national level pedometry data analyzed by other demographic variables are not yet available. In Colorado, walking, as measured with a Yamax SW-200 pedometer declined with age, was greatest in single men and women, was highest in respondents with incomes from $25-99,000 [48]. Lack of consistent study designs, measurement modalities, and reporting schemes makes it hard to generalize about walking/bicycling in different geographic areas. Comprehensive and objectively measured data addressing walking distance and duration might be required to fully describe age related changes in propensity to walk and the characteristics of walking trips.

### Strengths and limitations

Major strengths of this study include 1) our development of aggregate measures of street connectivity using principal components analysis of multiple aspects of connectivity, 2) Use of a multivariate model that is a mixture of logistic and lognormal regression components for the probability that a person walked and the amount walked, respectively, and 3) Explicit analysis of the spatial scale of street connectivity and AT implemented by running multivariate analyses at two geographic levels: zip code (large) and latitude/longitude (small). Together all three of these analytical approaches are advances over past studies. In particular, use of the multivariate model allows estimation of common effects of covariates on both propensity and duration and properly accounts for spatial autocorrelation. Residual analysis demonstrates that the model covariates explained all but the most local spatial effects in the original data.

There are at least four major weaknesses of the current study. First, this is a cross-sectional data set and so there are several possible alternatives to a simple causal relationship between connectivity and AT. Most notably, recent work suggests that self selection into neighborhoods with desirable features such as walkability, by people with a preference for walking could account for as much variation in walking as causal associations between neighborhood characteristics and walking [[10] p. 112,49,50]. Second, we were unable to obtain some important data elements in this project, specifically more comprehensive measures of land use mix. Land use mix is believed to be an important correlate of transportation walking and our use of employment density as a partial proxy for land use mix is not optimal. Ideally parcel level data on the use of different building would be collected, summarized in an index of mixed use and included in the kinds of models described here [51]. Recent examples of this approach [51,52], use square footage in three or more land use types such as residential commercial and office, in indices of walkability or the built environment. Such studies have reported positive associations between walking and walkability [45,52], but do not always attempt to separate the effects of connectivity, land use mix and other aspects of the built environment. Decomposition of these effects could increase the use of such studies by policy makers and urban planners [53].

CHIS 2001 queried respondents concerning walking and bicycling for transportation. While use of both modes represents 'active transportation', we acknowledge that walking and bicycling involve different skills, equipment, rewards, and infrastructure [54]. It seems likely that most of the active transportation examined in this study was due to walking and our examination of 'street connectivity' is arguably more relevant to walking. However, separate measures of walking and bicycling and examination of environmental features specifically related to walking vs. bicycling could strengthen and refine future studies. Later versions of CHIS have chosen to focus on walking, with separate questions concerning leisure and transportation walking as well as statewide geocoding http://www.chis.ucla.edu/.

Walking and bicycling use networks of roads, paths and sidewalks in different ways from each other and from automobiles. The present paper is entirely based on street networks. A few recent studies have contrasted the effects of pedestrian network analysis versus street network analysis on walking [55,56]. These two papers suggest that analysis of pedestrian networks can identify stronger and novel associations between network characteristics and pedestrian behavior than the analysis of street networks. CHIS data and further work to collect and analyze pedestrian network data from California could add to this promising research area.

The magnitude of the associations between street connectivity and AT observed in this study and others may seem small [30-33]. However, street connectivity is a modifiable feature of the environment and for a population with low levels of physical activity and high levels of sedentary behavior such as that of the United States [4,57], even small increases in physical activity could have significant population and individual health benefits [58].

## Conclusions

This paper significantly advances the analysis of street connectivity and AT by first identifying dominant axes from multiple measures of connectivity, using mixture models for the joint analysis of active transportation propensity and duration, and thirdly by explicitly examining spatial autocorrelation in the street connectivity variables and accounting for this variation in our analysis. Together the results indicated that aggregate measures of street connectivity are statistically significant correlates of AT independent of a number of individual and neighborhood characteristics. This result should encourage planners and policy makers interested in influencing physical activity for health, but also provide a cautionary note concerning the magnitude of expected effects.

## Competing interests

The authors declare that they have no competing interests.

## Authors' contributions

JD extracted the street connectivity variables and contributed to writing the paper. LP participated in the design of the study, performed the regression analyses and contributed extensively to writing the paper. DB conceived of the study, contributed to the statistical analyses, wrote the first draft of the manuscript, and integrated comments and text from the other authors and reviewers. All authors read and approved the final manuscript.'

## Supplementary Material

Additional file 1 **Regression coefficients from multivariate spatial analysis**. Regression coefficients from multivariate spatial analysis of the association between street connectivity, individual and neighborhood characteristics and active transportation. To address these goals we analyzed street connectivity and its association with AT using a large spatially identified data set collected as part of the 2001 California Health Interview Survey. Street connectivity represents a major class of environmental variables of great interest to health geographers because they are potentially correlated with multiple health behaviors and organized over diverse spatial scales. Click here for file
