# Supplementary material for: Associations between street connectivity and active transportation
Source: Int J Health Geogr. 2010 Apr 23;9:20. doi: 10.1186/1476-072X-9-20 (PMC2876088; doi:10.1186/1476-072X-9-20)
Supplement: Additional file 1 — Regression coefficients from multivariate spatial analysis. Regression coefficients from multivariate spatial analysis of the association between street connectivity, individual and neighborhood characteristics and active transportation. To address these goals we analyzed street connectivity and its association with AT using a large spatially identified data set collected as part of the 2001 California Health Interview Survey. Street connectivity represents a major class of environmental variables of great interest to health geographers because they are potentially correlated with multiple health behaviors and organized over diverse spatial scales. [file 1476-072X-9-20-S1.DOC]

| Additional Files  Regression coefficients from multivariate spatial analysis  Description: Regression coefficients from multivariate spatial analysis of the association between street connectivity, individual and neighborhood characteristics and active transportation. | | | | | | | | | | | | | |
| --- | --- | --- | --- | --- | --- | --- | --- | --- | --- | --- | --- | --- | --- |
|  | **Los Angeles** | | | | | | **San Diego** | | | | | | |
|  | Common coefficients | | Coefficients for | | | | Common coefficients | | | Coefficients for | | | |
|  | duration & propensity | | duration only | | propensity only | | duration & propensity | | | duration only | | propensity only | |
| Variable | Estimate | p | Estimate | p | Estimate | p^1^ | Estimate | | p | Estimate | p | Estimate | p^1^ |
| **Intercept** |  |  |  |  |  |  |  | |  |  |  |  |  |
| Propensity to walk | 0.6309 | 0.0275 |  |  |  |  | 0.6106 | | 0.0571 |  |  |  |  |
| Duration | 4.1506 | <.0001 |  |  |  |  | 4.5132 | | <.0001 |  |  |  |  |
|  |  |  |  |  |  |  |  | |  |  |  |  |  |
| **Individual Characteristics** |  |  |  |  |  |  |  | |  |  |  |  |  |
| Age effect by poverty level |  |  |  |  |  |  |  | |  | 0.0088 | 0.0030 | -0.0107 | <.0001 |
| poverty level <100% |  | 0.0003^2^ | 0.0008 |  | -0.0216 |  |  | |  |  |  |  |  |
| poverty level 100-200% |  | 0.0003^2^ | 0.0023 |  | -0.0201 |  |  | |  |  |  |  |  |
| poverty level 201-300% |  | 0.7894^2^ | 0.0113 |  | -0.0111 |  |  | |  |  |  |  |  |
| poverty level >300% |  | referent | 0.0106 | <.0001 | -0.0118 | <.0001 |  | |  |  |  |  |  |
| Female (Ref. = Male) | 0.0124 | 0.6989 |  |  |  |  | -0.0156 | | 0.8271 |  |  |  |  |
| Race (Ref. = NH White)^4^ |  |  |  |  |  |  |  | |  |  |  |  |  |
| Non-Hispanic Black | -0.1559 | 0.1358 |  |  |  |  | -0.0477 | | 0.7760 |  |  |  |  |
| Hispanic | 0.2350 | <.0001 |  |  |  |  | 0.1434 | | 0.1849 |  |  |  |  |
| Other | -0.0789 | 0.1319 |  |  |  |  | -0.1090 | | 0.3944 |  |  |  |  |
| BMI Kg-m^2^; (Ref. = 18.5-24.9) |  |  |  |  |  |  |  | |  |  |  |  |  |
| <18.5 | -0.1648 | 0.1275 |  |  |  |  | -0.4864 | | 0.0641 |  |  |  |  |
| 25.0-29.9 | -0.0552 | 0.1217 |  |  |  |  | -0.0267 | | 0.7351 |  |  |  |  |
| >=30.0 | -0.1015 | 0.0225 |  |  |  |  | 0.0277 | | 0.7823 |  |  |  |  |
| Health Limits (Ref = not at all) |  |  |  |  |  |  |  | |  |  |  |  |  |
| 1 Limited a lot |  |  | -0.2729 | 0.0005 | -0.6240 | 0.0021 | -0.5816 | | <.0001 |  |  |  |  |
| 2 Limited a little |  |  | -0.1555 | 0.0015 | -0.2015 | 0.5302 | -0.3801 | | <.0001 |  |  |  |  |
| Birthplace (Outside US) | 0.1254 | 0.0219 |  |  |  |  | 0.4087 | | 0.0044 |  |  |  |  |
| Education (Ref. = High School) |  |  |  |  |  |  |  | |  |  |  |  |  |
| <HS |  |  | -0.0175 | 0.7992 | 0.3166 | 0.0012 | -0.0609 | | 0.6632 |  |  |  |  |
| >HS (4) |  |  |  |  |  |  | 0.0259 | | 0.7579 |  |  |  |  |
| Blacks |  |  | -0.1293 | 0.3665 | -0.0438 | 0.0978 |  | |  |  |  |  |  |
| NH Whites, Hispanics, Others |  |  | -0.0869 | 0.1154 | 0.3311 | <.0001 |  | |  |  |  |  |  |
| Poverty level (Ref = >300%) |  |  |  |  |  |  |  | |  |  |  |  |  |
| <100% | 0.9036 | <.0001 |  |  |  |  | 0.1241 | | 0.3583 |  |  |  |  |
| 100-200% | 0.6301 | <.0001 |  |  |  |  | 0.2538 | | 0.0082 |  |  |  |  |
| 201-300% | 0.1247 | 0.3332 |  |  |  |  | 0.1149 | | 0.2666 |  |  |  |  |
|  |  |  |  |  |  |  |  | |  |  |  |  |  |
| Working now (Ref. = not working) |  |  |  |  |  |  | -0.3893 | | <.0001 |  |  |  |  |
| Blacks |  | 0.0071^3^ | -0.0605 |  | -0.4781 |  |  | |  |  |  |  |  |
| NH Whites, Hispanics, Others |  |  | -0.1941 | <.0001 | -0.6117 | <.0001 |  | |  |  |  |  |  |
|  |  |  |  |  |  |  |  | |  |  |  |  |  |
| Years in the US | -0.0009 | 0.6530 |  |  |  |  | -0.0118 | | 0.0322 |  |  |  |  |
|  |  |  |  |  |  |  |  | |  |  |  |  |  |
| **Neighborhood Characteristics** |  |  |  |  |  |  |  | |  |  |  |  |  |
| Population Density (Ref > 6348) |  |  |  |  |  |  |  | |  |  |  |  |  |
| 1 (1-2034) | -0.1299 | 0.0592 |  |  |  |  | -0.0685 | | 0.7260 |  |  |  |  |
| 2 (2035-3081) | -0.1392 | 0.0119 |  |  |  |  | -0.1014 | | 0.5620 |  |  |  |  |
| 3 (3082-4285) | -0.2306 | <.0001 |  |  |  |  | -0.0568 | | 0.7235 |  |  |  |  |
| 4 (4286-6348) | -0.0839 | 0.0630 |  |  |  |  | 0.0071 | | 0.9620 |  |  |  |  |
| Employment Density (Ref >2030) |  |  |  |  |  |  |  | |  |  |  |  |  |
| 1 (1-401) |  |  | 0.0520 | 0.4828 | -0.3323 | 0.0004 |  | |  | 0.0842 | 0.2506 | -0.7113 | <.0001 |
| 2 (402-779) |  |  | 0.1902 | 0.0039 | -0.3059 | <.0001 |  | |  | 0.2051 | 0.0018 | -0.9926 | <.0001 |
| 3 (780-1210) |  |  | 0.0743 | 0.2375 | -0.1853 | 0.0075 |  | |  | 0.0780 | 0.2133 | -0.6836 | 0.0001 |
| 4 (1211-2030) |  |  | -0.0151 | 0.8028 | -0.1600 | 0.1236 |  | |  | -0.0083 | 0.8910 | -0.8164 | <.0001 |
|  |  |  |  |  |  |  |  | |  |  |  |  |  |
| Bus Stop (1+) | 0.0411 | 0.5216 |  |  |  |  |  | |  | -0.2786 | 0.0285 | 0.2635 | 0.0025 |
| Freeway | 0.1101 | 0.0043 |  |  |  |  | not included in model | | |  |  |  |  |
| Bus Route | -0.1567 | 0.0217 |  |  |  |  | not included in model | | |  |  |  |  |
| Rail | 0.0430 | 0.4957 |  |  |  |  | not included in model | | |  |  |  |  |
|  |  |  |  |  |  |  |  |  | |  |  |  |  |
| **Street Connectivity** |  |  |  |  |  |  |  |  | |  |  |  |  |
| PRIN 1 |  |  | 0.0216 | 0.0842 | 0.0685 | 0.0150 | 0.0667 | 0.0019 | |  |  |  |  |
| PRIN 2 | 0.0258 | 0.0591 |  |  |  |  | 0.0361 | 0.1227 | |  |  |  |  |
|  |  |  |  |  |  |  |  |  | |  |  |  |  |
| Notes:  "Ref" denotes the referent group.  The p values shown are the results of a Type III Sums of Squares F test of how much the sum of squared residuals is reduced by this covariate when all other covariates are in the model, i.e., how important is this covariate for predicting AT, with the following exceptions:  ^1^The p value shown in the Propensity column indicates the significance of the **difference** between the duration and propensity effects.  ^2^ p value measures significance of difference between age effects for this poverty level and >300% level. Interaction effects (not shown separately) are included in duration and propensity estimates.  ^3^ p value measures significance of difference between work status effects for Blacks and all other race/ethnicity groups. Interaction effects (not shown separately) are included in duration and propensity estimates.  ^4^ Note that the effects shown by race here do not include any effects of work status since the effects of working and education were independent in the model. However, a complete assessment of patterns among Blacks should take both working and education into account. | | | | | | | | | | | | | |
